# Relationship between parenting stress profiles, work-family conflict, and adolescent problem behavior: Variable-centered and person-centered approaches

**DOI:** 10.1371/journal.pone.0340958

**Published:** 2026-02-25

**Authors:** Ying Yang

**Affiliations:** School of Marxism, Changchun Normal University, Changchun, China; Fakultet za pravne i poslovne studije dr Lazar Vrkatic, SERBIA

## Abstract

**Background:**

Adolescent problem behaviors, including both internalizing (e.g., emotional symptoms) and externalizing difficulties (e.g., conduct problems, hyperactivity), are a significant risk factor in adolescent development. This study assessed the impact of maternal parenting stress on adolescent problem behaviors and explored mechanisms underlying this relationship.

**Objective:**

This study aimed to (1) identify potential profiles of maternal parenting stress, (2) examine differences in adolescent problem behaviors across these profiles, and (3) determine whether maternal work-family conflict mediates the relationship between maternal stress profiles and adolescent problem behaviors.

**Methods:**

In this cross-sectional study, data were collected from 846 mother-child subject pairs through self-report instruments completed by both mothers and adolescents. Standardized measures were used to assess parenting stress, work-family conflict, and problem behavior. The data were subsequently analyzed using latent profile analysis and mediation analysis.

**Result:**

(1) Four maternal stress profiles were identified: low-stress, middle-stress-low interaction disorder, middle-stress, and high-stress profiles. (2) Significant differences in adolescent problem behaviors were observed across these profiles. (3) The other three profiles significantly predicted adolescent problem behaviors compared to the low-stress group. (4) Using the low-stress type as the reference, maternal work interference with family significantly mediated the relationship between the remaining three stress profiles and adolescent problem behaviors.

## 1. Introduction

Adolescent problem behavior refers to maladaptive behaviors that hinder adolescents’ social adjustment [1]. Such behaviors become more prevalent during adolescence, a period of rapid developmental and social change [2]. They can seriously impair adolescents’ physical and mental health and lead to difficulties in adjustment across home, school, and community contexts [3,4]. Typical manifestations include aggression, rule-breaking, and social withdrawal [5,6]. The family, as the most proximal environmental context, plays a critical role in adolescents’ development and adaptation [7]. In the Chinese cultural context, shaped by the traditional expectation that “children should have a bright future” and intense academic and occupational competition, parents often experience considerable pressure related to childrearing [8]. Understanding how mothers’ parenting stress is linked to adolescents’ problem behaviors in this context is therefore essential for promoting adolescents’ physical and mental health.

### 1.1. Heterogeneity in the parenting stress of mothers

Most existing studies have examined mothers’ parenting stress at the variable level. This approach can reflect overall differences and explain the interrelationships between mothers’ parenting stress and other variables. However, it assumes that all study subjects are homogeneous, which makes it challenging to capture individual differences fully and overlooks the heterogeneity of mothers’ parenting stress. Person-centered approaches such as latent profile analysis are specifically designed to capture such heterogeneity, in contrast to traditional variable-centered approaches [9–11]. These methods classify individuals into subgroups based on similar response patterns and examine whether distinct subgroups exist within the overall sample [12,13]. Individuals in the same group show similar psychological or behavioral patterns, whereas those in different groups differ significantly, which makes the results closer to real-world scenarios [14]. For example, a study of 6,021 mothers of children aged 3–5 years identified five parenting-stress profiles: low, moderate, high child-side, high parent-side, and high combined stress [15]. Another study of parents of infants and toddlers identified three profiles: low stress, middle stress, and high stress [16]. Taken together, these findings suggest that parenting stress among parents is heterogeneous and can be meaningfully grouped into distinct patterns rather than treated as a single homogeneous construct. Therefore, the present study used latent profile analysis to identify distinct profiles of parenting stress among mothers.

### 1.2. The relationship between mothers’ parenting stress profiles and adolescent problem behavior

Maternal parenting stress refers to the stressful experience of mothers who struggle to meet the expectations and demands of their families and children due to the limited nature of their personal and social resources [17]. In the Chinese cultural context, where mothers are typically the primary caregivers, they tend to experience higher levels of parenting stress than fathers [18,19]. Such stress can impair mothers’ physical and mental health, undermine their parenting efforts, and reduce the quality of family life, which in turn affects children’s development [19].

According to the spillover hypothesis of family systems theory, the emotional feelings experienced and stress generated by individuals in the couple subsystem may spill over into the parent-child subsystem [20]. High-intensity parenting stress often migrates to parenting behaviors and emotional expression, impairing parents’ ability to engage in positive parenting behaviors and causing parents to develop negative thoughts or behaviors [21], such as alienation, avoidance responses to children, and varying degrees of child abuse [22]. It affects parents’ emotional and behavioral support functions for their children and leads to unhealthy family environments [23]. Children in such high-stress environments may internalize the stress they experience, which can manifest as internalizing problems, such as heightened anxiety symptoms [24]. Empirical studies have also shown that higher levels of maternal parenting stress predict more externalizing behavior problems in children (e.g., aggression, rule-breaking) [25], rather than internalizing symptoms [26].

The effects of different types of parenting stress on mothers’ and children’s problem behaviors can vary. For example, one study identified five trajectories of maternal parenting stress, including persistently low, persistently mild, moderately decreasing, moderately increasing, and persistently high [27]. Adolescents whose mothers followed any trajectory other than the persistently low group showed higher levels of internalizing symptoms, and similar patterns were found for externalizing symptoms [27]. New parenting demands are placed on parents for adolescents during a rapid phase of development and change [28]. Therefore, examining the relationship between parenting stress and adolescent problem behaviors among mothers with different types of stress will provide theoretical guidance for proposing more targeted interventions to alleviate adolescent problem behaviors.

### 1.3. The mediating role of mothers’ work-family conflict

Work-family conflict is a type of conflict that arises when an individual is unable to simultaneously meet the demands of two different roles, work and family [29]. In the Chinese context, mothers’ high labor-force participation combined with traditional gender roles means that they shoulder most family responsibilities and thus experience particularly salient work–family conflicts [29,30]. With the implementation of China’s ‘two-child’ and ‘three-child’ policies, many working mothers must devote even more time and energy to childcare while maintaining their careers and providing financial support for the family [31,32]. In addition, with the advancement of technology and the widespread adoption of communication tools, the current popular telecommuting model blurs the boundaries between an individual’s work and home life [33]. Work continues to encroach on the time and energy individuals devote to their families, affecting communication and interaction among family members [34].

Although prior studies have shown an association between maternal parenting stress and adolescents’ problem behaviors, the mechanisms underlying this association remain unclear, partly because potential mediating factors have received limited attention. The Cumulative Risk Model states that psychological and behavioral problems in children’s physical and mental development are caused by the persistent influence of external negative risk factors [35], of which work-family conflict cannot be ignored [36]. When the demands placed on the individual by the work and family spheres are inconsistent, the individual’s family resources are threatened or even eliminated [37]. Therefore, further examining whether and how mothers’ work–family conflict mediates the link between parenting stress and adolescent problem behaviors may help clarify this mechanism and inform strategies to mitigate its adverse effects.

Role conflict between work and family is strongly associated with parenting stress. The Work-Home Resources Model posits that work-family conflict arises when the demands of the work domain consume personal resources, thereby preventing individuals from contributing to the family domain [38]. Consistent with the matching hypothesis, such conflict has particularly strong effects on family-related outcomes [32]. It has been shown that work-family conflict reduces mothers’ parenting involvement, diminishes their ability to cope with parenting stress [39], and increases mothers’ stress and emotional depletion during interactions with their children [29,40]. Taken together, these findings indicate that women who experience higher levels of work–family conflict are more likely to report elevated parenting stress.

Factors contributing to adolescent problem behavior may be influenced by the mother’s work-family conflict and parenting pressures from the mother. Research has found that work-family conflict diverts time and emotional resources away from parents, requiring them to deal with their children’s problems [41]. At the same time, parents caught in work–family conflicts are prone to lose patience in parenting and to avoid parenting responsibilities [42]. They may neglect their children’s psychological needs and adopt more harsh parenting styles, such as scolding and criticizing [43]. These parents are also more likely to transfer the negative emotions aroused by work–family conflicts into parent–child interactions, which undoubtedly increases the risk of adolescent problem behaviors [44]. In the Chinese context, although the work environment is not significantly different from that of the West, Chinese employees tend to prioritize their families due to stronger family values and focus more on balancing family interests when dealing with work-family relationships [45]. Therefore, it is necessary to explore the mediating mechanisms of work-family conflict between maternal parenting stress and adolescent problem behaviors in the Chinese context.

### 1.4. The present study

Based on a large sample of surveyed adolescents and their mothers, this study uses latent profile analysis to identify distinct latent profiles of mothers’ parenting stress in the Chinese context and to examine their associations with adolescent problem behaviors. The study also incorporates the sub-dimensions of mothers’ work–family conflict to construct a concurrent mediation model and proposes the following hypotheses:

**Hypothesis 1.** Consistent with previous person-centered studies, maternal parenting stress will cluster into multiple latent profiles, including at least a low-stress profile, a moderate-stress profile, and one or more high-stress profiles.

**Hypothesis 2.** Maternal latent parenting stress profiles will significantly predict adolescents’ problem behaviors, with higher-stress profiles being associated with higher levels of adolescent problem behaviors than the low-stress profile.

**Hypothesis 3.** The sub-dimensions of maternal work-family conflict mediate the relationship between maternal latent stress profiles and adolescent problem behavior.

## 2. Methods

### 2.1. Participants and procedure

In this cross-sectional study, high school students and their mothers were recruited through convenience sampling from September 15 to November 5, 2024, in Northeast China, with a total sample size of 921 individuals. Seventy-five subjects were excluded due to mis-filling, omission, or irregularity in the answers to the questions. The remaining 846 adolescents and their mothers were included in the study, resulting in an effective participation rate of 91.86%. The accessibility of the target population within the local school system, combined with practical constraints of time and resources, justified the use of a convenience sampling method. This approach has been widely applied in educational and psychological research when the primary aim is to examine mechanisms rather than to achieve strict population representativeness. Moreover, the final adequate sample of 846 dyads ensured sufficient statistical power for the planned analyses. Prior methodological studies indicate that sample sizes above 500 are adequate for latent profile analysis and mediation models [46,47]. Compared with similar studies that typically involve 300–500 participants, the present sample is considerably larger, thus providing reliable and stable estimates. Sociodemographic characteristics are summarized below and detailed in Table 1.

**Table 1:** Participant characteristics (*N* = 846).

Demographic characteristic		*N*	Proportion (*%*)
Adolescent Gender	Boy	416	49.17
	Girl	430	50.83
Number of children in a family	One child	274	32.39
	Two children and above	572	67.61
Maternal Age	35-40 years old	95	11.23
	41-45 years old	592	69.98
	46-50 years old	159	18.79
Educational Level of Mothers	Secondary school and below	314	37.12
	College or correspondence undergraduate	199	23.52
	Full-time undergraduate degree	297	35.11
	Master’s degree or above	36	4.26
Family Structure	Divorced or single-parent families	86	10.17
	Nuclear family	515	60.87
	The big family of three generations	219	25.89
	Other family forms	26	3.07
Working Hours	Full-time mothers	162	19.15
	8 hours or less	252	29.79
	8 hours or more	432	51.06
Month Income	RMB 6,000 and below	186	21.99
	RMB 6,000–10,000	265	31.32
	RMB 10,000–15,000	194	22.93
	RMB 15,000–20,000	100	11.82
	RMB 20,000 and above	101	11.94

First, mothers of high school subjects were provided with a link to open an online consent form, which described the subjects’ rights and informed them that the relevant data would be used only for the study, that participation in the study was voluntary, and that refusal to participate and withdrawal from the study would not result in adverse consequences. Mothers then decided whether to consent to participate in the study. Surveys were offered only if mothers agreed to participate. Mothers’ demographic characteristics (e.g., age) were first surveyed, and then mothers were surveyed about their levels of parenting stress and work-family conflict. For adolescents, with parental consent, paper problem behavior questionnaires were administered to high school students in a school setting. All study procedures complied with the ethical principles of the WMA Declaration of Helsinki. The study protocol was reviewed and approved by the Ethics Committee of our university (approval number ME2024−45).

### 2.2. Measures

#### 2.2.1. Parenting stress.

Maternal parenting stress was assessed using the Parenting Stress Index–Short Form (PSI-SF) [48], which has been revised for use in Chinese populations [49]. The PSI-SF consists of 36 items rated on a 5-point Likert scale ranging from 1 (strongly disagree) to 5 (strongly agree), with higher scores indicating greater levels of parenting stress. The scale comprises three subscales—Parental Distress, Parent–Child Dysfunctional Interaction, and Difficult Child—with 12 items in each subscale. The parental distress dimension reflects stress caused by parents’ personal factors, such as role restriction, conflicts with a partner, and lack of social support (e.g., “I feel that being a parent restricts me”). The parent-child dysfunctional interaction dimension captures parents’ perceptions of disappointment in interactions with their child and feelings of alienation (e.g., “Many times I feel that my child does not like me and does not want to be close to me”). The difficult child dimension assesses parents’ perceptions that the child has demanding temperamental characteristics, such as high activity level or emotional intensity, which increase parenting burden (e.g., “My child seems to cry more often than most children”). The Cronbach’s alpha coefficients were 0.918 for parental distress, 0.885 for parent-child dysfunctional interaction, and 0.806 for difficult child. A three-factor confirmatory factor analysis (CFA) conducted in the present sample supported the expected structure of the PSI-SF, *χ*^*2*^*/df* = 6.238, TLI = 0.817, CFI = 0.828, RMSEA = 0.079, SRMR = 0.074.

#### 2.2.2. Problem behavior.

This study used the Strengths and Difficulties Questionnaire (SDQ) [50]. The Chinese version of the SDQ has shown good reliability and validity in research involving Chinese populations [51]. The SDQ contains 25 items across five subscales. In this study, we used the four difficulties subscales, each comprising five items. Emotional symptoms (internalizing manifestations such as persistent worries or somatic complaints; e.g., “often feels nervous or worried”), conduct problems (rule‑breaking and oppositional behaviors; e.g., “often gets into fights or breaks rules”), hyperactivity/inattention (restlessness and difficulty sustaining attention; e.g., “restless, overactive, cannot stay still for long”), and peer problems (difficulties with peer relationships; e.g., “tends to play alone/ has few close friends”). Items were rated on a 3‑point Likert scale from 0 = not true, 1 = somewhat true, to 2 = certainly true; reverse‑scored items were recoded so that higher scores indicate greater difficulties. Subscale scores were summed, and a total difficulties score (sum of the four subscales; 20 items) was computed, excluding prosocial behavior. The Cronbach’s alpha coefficients were 0.627 for emotional symptoms, 0.692 for conduct problems, 0.635 for hyperactivity, and 0.672 for peer problems. The overall problem behavior scale demonstrated acceptable internal consistency (α = 0.715), whereas the internal consistency of the 5-item subscales was in the moderate range. A four-factor confirmatory factor analysis (CFA) conducted in the present sample supported the expected structure of the four difficulties subscales, *χ*^*2*^*/df* = 4.378, TLI = 0.726, CFI = 0.764, RMSEA = 0.063, SRMR = 0.020.

#### 2.2.3. Work-family conflict.

This study used the Work-Family Conflict Scale [52], which has been revised for Chinese samples [53]. The scale includes two dimensions: Work Interfering with Family (WIF), which refers to situations where work demands negatively affect family responsibilities, and Family Interfering with Work (FIW), which refers to situations where family demands hinder work performance. Each dimension consists of 9 items, for a total of 18 items. Responses were rated on a 5-point Likert scale ranging from 1 = strongly disagree to 5 = strongly agree, with higher scores indicating greater levels of work–family conflict. Representative items include: for WIF, “Because of work, I have to miss some family activities”; and for FIW, “I often feel so burdened by family responsibilities that it is difficult to concentrate on my work.” In the present sample, the Cronbach’s alpha coefficients were 0.872 for WIF and 0.885 for FIW. The overall scale demonstrated excellent internal consistency, with a Cronbach’s alpha of 0.933. A two-factor confirmatory factor analysis (CFA) conducted in the present sample supported the expected structure of the two dimensions, *χ*^*2*^*/df* = 7.729, TLI = 0.843, CFI = 0.862, RMSEA = 0.085, SRMR = 0.047.

### 2.3. Data analysis

Raw data were entered and preliminarily analyzed using SPSS 26.0. To examine potential methodological bias caused by self-report measures, Harman’s one-way test was conducted to test for common method variance [54,55]. In the first step, confirmatory factor analyses (CFAs) were conducted to examine the measurement structure of the study scales. Model fit was evaluated using *χ²/df*, CFI, TLI, RMSEA, and SRMR, following commonly recommended criteria [56,57]. Next, descriptive statistics, independent-samples t tests, and one-way ANOVA were used to examine differences in adolescent problem behaviors across demographic variables. Correlation analyses were also performed to assess the relationships among maternal parenting stress, work-family conflict, and adolescent problem behaviors, providing the basis for subsequent analyses.

In the second step, latent profile analysis (LPA) was performed using Mplus 8.0 to identify potential profiles of maternal parenting stress. As a person-centered approach, LPA classifies individuals with similar response patterns into the same latent profile on a probabilistic basis [58]. By comparing the fit indices of different models, this method helps determine the optimal solution and maximizes between-profile heterogeneity and within-profile homogeneity [59]. Missing values were interpolated using the maximum likelihood method. Model fit was evaluated using a range of indices, including the Bayesian Information Criterion (BIC) and sample size–adjusted BIC (aBIC), with smaller values indicating better model fit [60,61]. According to methodological studies [62–64], BIC and aBIC are considered more stable and reliable indicators for class enumeration; therefore, they were primarily emphasized in this study. Information entropy was also calculated, with values closer to 1 indicating more accurate classification, and a threshold of 0.80 was considered acceptable [65,66]. In addition, classification quality was further evaluated using average posterior probabilities (AvePP) for each profile, with values above the recommended threshold of 0.80 indicating good classification accuracy [62,64,67]. Moreover, the Lo–Mendell–Rubin likelihood ratio test (LMRT) and the Bootstrap likelihood ratio test (BLRT) were applied, with significant p values suggesting that a k-class model provides a better fit than a k-1 class model [68]. In addition to statistical criteria, interpretability and theoretical meaningfulness of the latent classes were also considered when determining the optimal model [69].

In the third step, before conducting group comparisons, we examined the assumptions of one-way ANOVA. Homogeneity of variances was assessed with Levene’s test. When this assumption was satisfied, we conducted one-way ANOVA with Tukey’s HSD post-hoc tests; when it was violated, Welch’s ANOVA with Games–Howell post-hoc tests was used. For all pairwise comparisons, Cohen’s d was calculated to quantify effect sizes. Based on the selected LPA model, one-way ANOVA was conducted to compare differences in adolescent problem behaviors among maternal parenting stress profiles. Linear regression was used to examine the associations between parenting stress profiles and adolescent problem behaviors, while controlling for relevant variables. Finally, mediation analyses were performed to test whether the dimensions of work–family conflict mediated the relationship between maternal parenting stress profiles and adolescent problem behaviors. Since the independent variable (parenting stress profiles) was categorical, relative mediation and overall mediation effects were estimated. When the dependent variable was continuous (adolescent problem behavior), mediation analyses were conducted using Model 4 of the PROCESS macro for SPSS (Hayes, 2013). The significance of indirect effects was tested with 5,000 bootstrap samples, and 95% confidence intervals were estimated.

## 3. Results

### 3.1. Common method deviation test

Because data collection using the self-report method may lead to common method bias [55], we used the Harman one-way test to test for common method bias after data collection was completed [54]. The results showed that the total number of factors with eigenvalues greater than 1 totaled 14, and the amount of variation explained by the first factor was 26.26%, which was less than the critical criterion of 40%, indicating that there was no significant common methodological bias in this study, allowing for further data analysis.

### 3.2. Correlations among variables

The means, standard deviations, and correlation coefficients of the study variables used in the subsequent analyses are shown in Table 2. The correlations among the dimensions of maternal parenting stress, work–family conflict, and adolescent problem behavior were all positive and statistically significant, indicating that these variables were appropriate for the latent profile, regression, and mediation analyses conducted in this study.

**Table 2:** Means, standard deviations, and correlations of all variables in this study (*N* = 846).

Variables	*M ± SD*	1	2	3	4	5	6	7
1 Adolescent Gender	–	–						
2 Parental Distress	30.25 *±*9.384	0.006	–					
3 Parent-Child Dysfunctional Interaction	22.68 *±*6.687	0.061	0.497***	–				
4 Difficult Child	30.02 *±*6.807	−0.013	0.563***	0.646***	–			
5 Adolescent Problem Behavior	9.03 *±*4.382	0.124***	0.322***	0.316***	0.422***	–		
6 Work-Interference-Family	24.33 *±* 6.970	0.003	0.612***	0.399***	0.423***	0.274***	–	
7 Family-Interference-Work	22.43 *±*6.831	0.011	0.685***	0.487***	0.469***	0.275***	0.840***	–

***Notes*:** Adolescent gender was dummy coded (1 = male, 0 = female); ^*^p < 0.05, ^***^p < 0.001; This table was created by the authors based on the study data.

### 3.3. Demographic differences in adolescent problem behavior scores

An analysis of the differences in adolescent problem behaviors across demographic variables revealed no significant differences in adolescent problem behaviors related to family structure and monthly family income. Boys had significantly higher levels of problem behavior than girls. Only children had significantly higher levels of problem behavior than non-only children. The level of problematic behavior of adolescents whose mothers were below 40 years of age was significantly higher than that of adolescents whose mothers were above 40 years of age. The level of problematic behaviors of adolescents whose mothers had less than a Bachelor’s degree was significantly higher than that of adolescents whose mothers had more than a Bachelor’s degree. The level of problematic behaviors among adolescents whose mothers were full-time mothers or worked more than 8 hours per day was significantly higher than that among adolescents whose mothers worked 8 hours or less per day. Specific results are presented in S1 and S2 Tables in S1 File. Based on these results, adolescent gender, number of children in the family, maternal age, mother’s educational level, and working hours were controlled as covariates in the subsequent analysis.

### 3.4. Results of a latent profile analysis of mothers’ parenting stress

Models were estimated with MLR, assuming local independence (within‑profile covariances fixed to zero) and equal residual variances across profiles, with profile means freely estimated. The results showed that the BIC and aBIC values gradually decreased as the potential profiles increased from 1 to 5, while the Entropy values exceeded 0.8 in profiles 3–5, indicating that profiles 3–5 all had high classification accuracy. Therefore, further comparisons must be made with the LMRT and BLRT indices. The results showed that the BLRT test value reached significance (*p* < 0.001) in all models, while the LMRT test value did not reach significance in model 5. In the 4-profile model, the proportion of the least profile group was 11.7%. Therefore, by combining the values of statistical parameters with the theoretical and practical significance of categorization, the 4-profile model was selected as the best model in this study. This means that there may be four profiles in the sample of mothers’ parenting stress, as shown in Table 3.

**Table 3:** Results of model fitting for latent profile analysis of mothers’ parenting stress.

Model	Loglikelihood	Free Parameters	BIC	aBIC	Entropy	LMRT(*p*)	BLRT(*p*)	Proportion Min
1	−8724.192	6	17488.827	17469.773	—	—	—	1.000
2	−8397.805	10	16863.015	16831.259	0.751	< 0.001	< 0.001	0.346
3	−8245.852	14	16586.070	16541.611	0.820	< 0.001	< 0.001	0.208
**4**	**−8151.183**	**18**	**16423.695**	**16366.532**	**0.876**	**< 0.001**	**< 0.001**	**0.117**
5	−8101.698	22	16351.688	16281.823	0.841	0.054	< 0.001	0.097

***Note:*** BIC = Bayesian Information Criterion; aBIC = Sample-size adjusted Bayesian Information Criterion; LMRT = adjusted Lo – Mendell – Rubin Likelihood Ratio Test; BLRT = Bootstrapped Likelihood Ratio Test; This table was created by the authors based on the study data.

**Fig 1:**
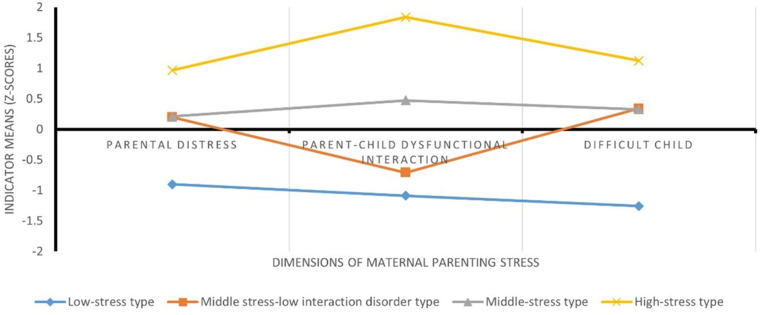
Profiles of mothers’ parenting stress. Indicator means are shown as z-scores across three dimensions: parental distress, parent–child dysfunctional interaction, and difficult child. Profiles: I = low-stress type; II = middle stress–low interaction disorder type; III = middle-stress type; IV = high-stress type.

As shown in Fig 1, the standardized z-scores of the four potential profiles of parenting stress among mothers (profiles 1–4, from bottom to top of the line) exhibited different characteristics on the dimensions of parental distress, parent-child dysfunctional interaction, and difficult children. Mothers in profile 1 accounted for 26.71% (*n* = 226) of the total number of mothers, and this group of mothers had the lowest levels of parenting stress in all dimensions, hence the name “low-stress type.” Mothers in profile 2 accounted for 18.32% (*n* = 155) of the total number of mothers. This group of mothers scored significantly higher than profile 1 in the parental distress and difficult child dimensions at a medium level. However, this group of mothers scored similarly to profile 1 in the parent-child dysfunctional interaction dimensions at a low level, so it was named the “middle stress-low interaction disorder type.”

Mothers in profile 3 accounted for 43.26% (*n* = 366) of the total number of mothers, and this group of mothers fell within the middle range for all dimensions of parenting stress, as well as for the overall level, hence the name “middle-stress type.” Mothers in profile 4 accounted for 11.70% (*n* = 99) of the total number of mothers, and this group of mothers had high levels of all dimensions of parenting stress, as well as overall levels, which were significantly higher than those in profile 3. It was, therefore, labeled as a “high-stress type.” The classification quality was high, with an entropy value of 0.876. The average posterior probabilities (AvePPs) for the most likely class membership were 0.947, 0.944, 0.859, and 0.963 for the four profiles, respectively, all exceeding the recommended threshold of 0.80. These values indicate that individuals were classified into their respective profiles with high precision, supporting the reliability of the identified 4-profile solution.

### 3.5. Mothers’ parenting stress profiles and adolescent problem behavior

**Fig 2:**
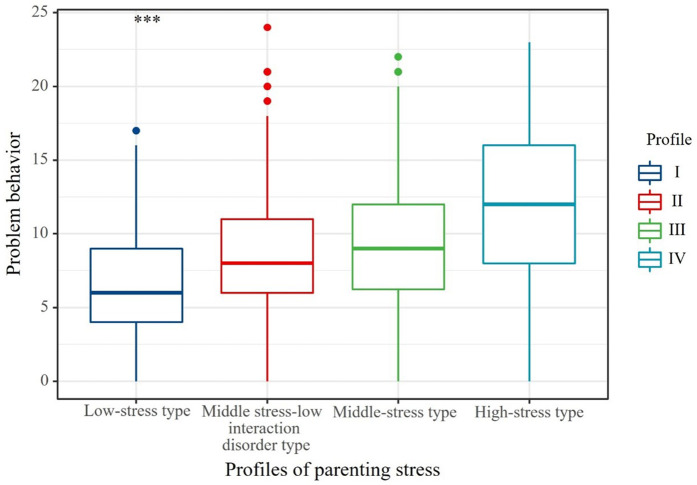
Differences in adolescent problem behavior across mothers’ parenting stress profiles. Notes: Boxes indicate the interquartile range with the median; whiskers indicate the range excluding outliers. ***p < 0.001. Profiles: I = low-stress type; II = middle stress–low interaction disorder type; III = middle-stress type; IV = high-stress type.

Prior to the distal comparisons, we examined the distinctness of the four profiles by testing equality constraints on the indicator means across classes using a Wald *χ²* test in Mplus, which indicated clear differences among profiles (Wald χ²(9) = 4667.10, *p* < .001). Using adolescents’ problem behavior as the distal outcome, Levene’s test of homogeneity of variances was significant, W (3, 842) = 9.71, *p* < .001, indicating that the assumption of equal variances was violated. Accordingly, we report the Welch robust test of mean differences, which showed a significant difference in problem behavior across the four profiles, F (3, 310.72) = 40.97, *p* < .001, *η²* = .128 (see Fig 2). Descriptively, mean (SD) scores were 6.85 (3.43) for Profile 1, 9.03 (4.09) for Profile 2, 9.55 (4.15) for Profile 3, and 12.05 (5.18) for Profile 4. Post-hoc pairwise comparisons using the Games–Howell procedure showed that adolescents with low‑stress mothers (Profile 1) scored significantly lower than those in Profiles 2–4 (all *p* < .001; Cohen’s *d* = 0.59–1.29). Adolescents with high‑stress mothers (Profile 4) scored significantly higher than those in Profiles 1–3 (all *p* < .001; Cohen’s *d* = 0.57–1.29). The difference between Profiles 2 and 3 was not significant (*p* = .538; *d* = 0.13). These findings indicate a graded pattern in which the high‑stress profile is associated with the highest level of problem behavior and the low‑stress profile with the lowest.

On the premise that both maternal parenting stress and each of its dimensions were significantly related to adolescent problem behavior, this study further explored the relationship between profiles of maternal parenting stress and adolescent problem behavior. Hierarchical multiple regression analyses were conducted to examine whether maternal parenting-stress profiles explained additional variance in adolescent problem behavior beyond demographic characteristics. In Model 1 (Step 1), adolescent gender, only-child status, maternal age, maternal educational level, and mothers’ working hours were entered as control variables, accounting for 8.4% of the variance in adolescent problem behavior (*R²* = 0.084, *F* = 8.479, *p* < .001). In Model 2 (Step 2), three dummy variables representing maternal parenting-stress profiles (high-stress, middle-stress, and middle-stress–low-interaction-disorder, with the low-stress profile as the reference group) were added. This significantly improved the model, yielding a significant increase in explained variance (Δ*R²* = 0.120, Δ*F* = 41.691, *p* < .001) and raising the total *R²* to 0.203 (*F* = 17.710, *p* < .001; see Table 4). In the final model, compared with adolescents whose mothers were in the low-stress profile, those whose mothers were in the high-stress, middle-stress, and middle-stress–low-interaction-disorder profiles showed significantly higher levels of problem behavior (*β* = 0.379, *t* = 10.707, *p* < .001; *β* = 0.298, *t* = 7.718, *p* < .001; *β* = 0.180, *t* = 4.907, *p* < .001). These findings indica*t*e tha*t* maternal paren*t*ing-stress profiles make a unique and positive contribution to adolescents’ problem behaviors over and above demographic characteristics.

**Table 4:** Results of regression analysis of mothers’ parenting stress latent profiles and adolescent problem behavior scores.

Model			*B*	*SE*	*β*	*t*	VIF
Common element			6.538	0.803	—	7.918***	—
Model 1	Adolescent Gender	Boy	1.005	0.291	0.115	3.459**	1.004
		Girl	0	—	—	—	—
	Number of Children in Family	One child	1.254	0.339	0.134	3.699***	1.197
		Two children and above	0	—	—	—	—
	Maternal Age	35-40 years old	2.121	0.574	0.153	3.698***	1.560
		41-45 years old	0.171	0.390	0.018	0.438	1.522
		46-50 years old	0	—	—	—	—
	Educational Level of Mothers	Secondary school and below	2.162	0.768	0.238	2.815**	6.546
		College or correspondence undergraduate	1.712	0.769	0.166	2.226*	5.063
		Full-time undergraduate degree	0.900	0.747	0.098	1.204	6.049
		Master’s degree or above	0	—	—	—	—
	Working Hours	Full-time mothers	0.256	0.429	0.023	0.596	1.354
		8 hours or less	-0.537	0.336	-0.056	-1.598	1.126
		8 hours or more	0	—	—	—	—
*R* ^*2*^		0.084	△*R*^*2*^		0.084		
*F*		8.479***	△*F*		8.479***		
Common element			4.625	0.770	—	6.005***	—
Model 2	Adolescent Gender	Boy	0.833	0.272	0.095	3.057**	1.011
		Girl	0	—	—	—	—
	Number of Children in Family	One child	1.401	0.317	0.150	4.418***	1.201
		Two children and above	0	—	—	—	—
	Maternal Age	35-40 years old	1.888	0.537	0.136	3.515***	1.569
		41-45 years old	-0.053	0.365	-0.006	-0.144	1.529
		46-50 years old	0	—	—	—	—
	Educational Level of Mothers	Secondary school and below	1.757	0.720	0.194	2.440*	6.594
		College or correspondence undergraduate	1.862	0.720	0.180	2.587*	5.079
		Full-time undergraduate degree	1.044	0.699	0.114	1.494	6.068
		Master’s degree or above	0	—	—	—	—
	Working Hours	Full-time mothers	0.078	0.401	0.007	0.193	1.358
		8 hours or less	-0.750	0.315	-0.078	-2.378*	1.133
		8 hours or more	0	—	—	—	—
	High-stress type		5.164	0.482	0.379	10.707***	1.311
	Middle-stress type		2.631	0.341	0.298	7.718***	1.555
	Middle-stress-low interaction disorder type		2.032	0.414	0.180	4.907***	1.399
	Low-stress type		0	**—**	—	—	—
*R* ^*2*^		0.203	△*R*^*2*^		0.120		
*F*		17.710***	△*F*		41.691***		
Dependent variable： Adolescent Problem Behavior							

**Notes:** *p < 0.05, **p < 0.01, ***p < 0.001; This table was created by the authors based on the study data.

**Abbreviations:** VIF, variance inflation factor.

### 3.6. The mediating role of mothers’ work-family conflict

Based on previous research, profiles of maternal parenting stress were virtually coded (D1: 0 = middle stress-low interaction disorder type, 0 = middle-stress type, 1 = high-stress type; D2: 0 = middle stress-low interaction disorder type, 1 = middle-stress type, 0 = high-stress type; D3: 1 = middle stress-low interaction disorder type, 0 = middle-stress type, 0 = high-stress type), and work-family conflict subdimensions were used as mediating variables and adolescent problem behavior as the dependent variable to build a multiple mediation model. The results showed that the overall total effects test of *F* (3, 842) = 41.691, *p* < 0.001, and the three relative total effects were not all zero; the overall direct effects test of *F* (3, 842) = 21.379, *p* < 0.001, and the three relative direct effects were not all zero, making further relative mediation analyses necessary.

**Fig 3:**
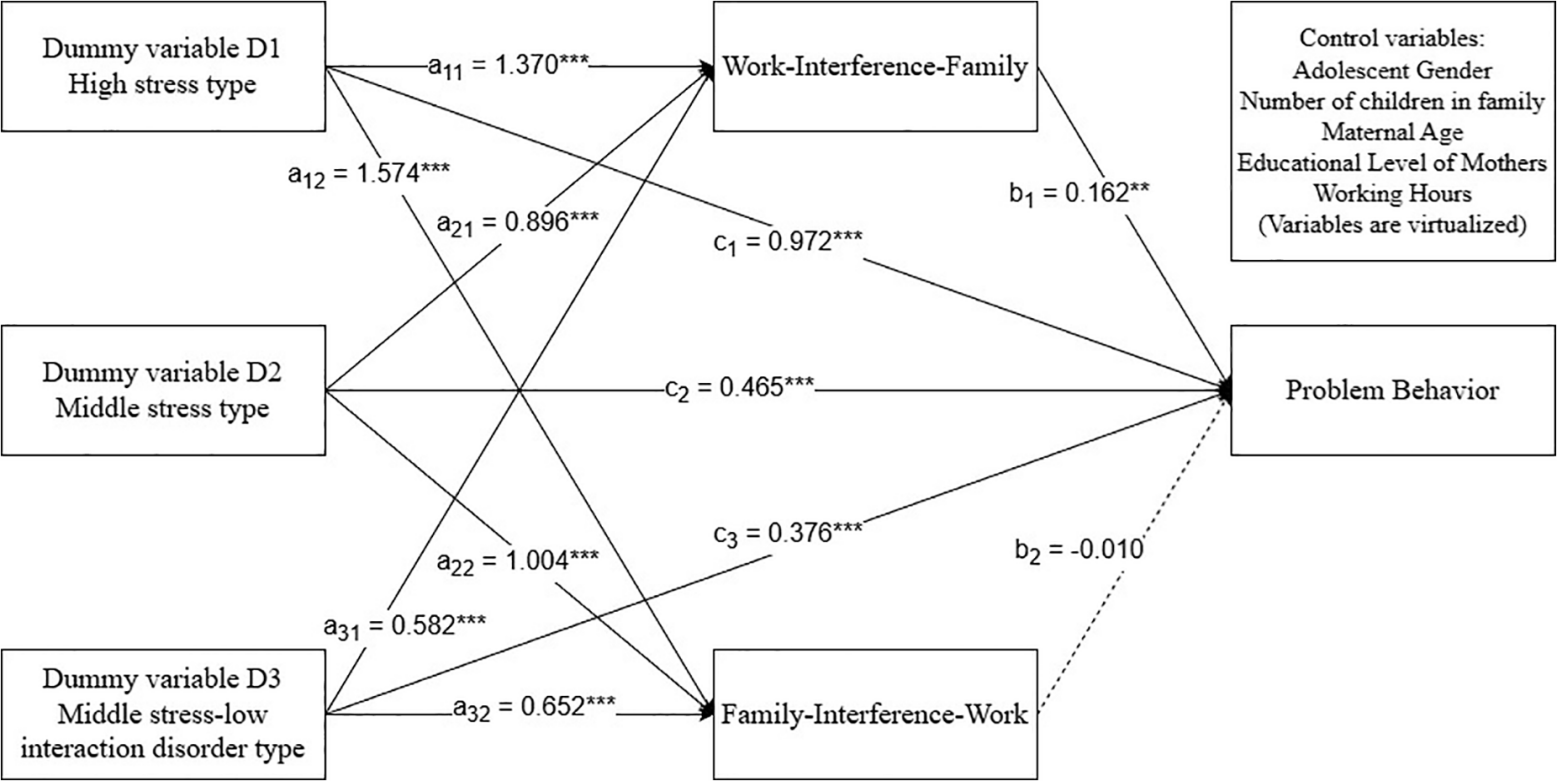
Mediated pathways linking mothers’ parenting stress profiles, work–family conflict, and adolescent problem behavior. Notes: Solid lines indicate significant paths; dashed lines indicate nonsignificant paths. Values on paths are standardized regression coefficients (β). The low-stress type served as the reference group. Models were adjusted for adolescent gender, number of children in the family, maternal age, mothers’ educational level, and working hours. **p < 0.01, ***p < 0.001.

Using the low‑stress profile as the reference group and controlling for covariates, only the Work‑Interference‑with‑Family (WIF) pathway showed significant mediation across profile contrasts. In contrast, the Family-Interference-with-Work (FIW) pathway was not significant. As shown in Table 5, WIF partially mediated the associations for high-stress vs. low-stress (indirect *β* = 0.222; relative mediated proportion = 18.83%), middle-stress vs. low-stress (indirect *β* = 0.145; 24.17%), and middle–stress–low–interaction vs. low-stress (indirect *β* = 0.094; 20.26%). Direct effects (c’) remained significant in all three contrasts, indicating partial (not complete) mediation. The FIW indirect effects were small and non‑significant across contrasts. Consistent with recommendations for multi-categorical mediation models, statistical inference for these relative effects was based on bias-corrected 95% bootstrap confidence intervals rather than conventional p values [70,71]; an effect was considered significant when its 95% bootstrap confidence interval did not include zero, and such effects are marked with asterisks in Table 5. Fig 3 presents the model.

**Table 5:** Multiple mediator model test results for adolescent problem behavior.

Groups	*β*	*SE*	LLCI	ULCI	Relative effect size
High-stress type					
Relative direct effect	0.972*	0.123	0.731	1.213	—
Relative mediating effect _ WIF	0.222*	0.084	0.068	0.404	18.83%
Relative mediating effect _ FIW	−0.015	0.097	−0.210	0.169	1.27%
Relative total effect	1.179*	0.110	0.963	1.395	—
Middle-stress type					
Relative direct effect	0.465*	0.085	0.298	0.632	—
Relative mediating effect _ WIF	0.145*	0.056	0.043	0.265	24.17%
Relative mediating effect _ FIW	−0.010	0.062	−0.132	0.109	1.67%
Relative total effect	0.600*	0.078	0.448	0.753	—
Middle-stress-low interaction disorder type					
Relative direct effect	0.376*	0.096	0.187	0.565	—
Relative mediating effect _ WIF	0.094*	0.039	0.026	0.182	20.26%
Relative mediating effect _ FIW	−0.006	0.040	−0.087	0.071	1.29%
Relative total effect	0.464*	0.095	0.278	0.649	—

**Notes:** All coefficients (*β*) are standardized regression coefficients obtained from the mediation model; Low-stress type as a reference group; Control variables, adolescent gender, number of children in the family, maternal age, mother’s educational level, and working hours; Relative effect sizes represent the proportion of the relative mediating effect in the total effect; Asterisks indicate effects whose 95% bootstrap confidence interval does not include zero; This table was created by the authors based on the study data.

**Abbreviations:** LLCI, the lower limit of confidence interval; ULCI, the upper limit of the confidence interval; WIF, Work-Interference-Family; FIW, Family-Interference-Work.

## 4. Discussion

### 4.1. Profiles of mothers’ parenting stress

The present study found that mothers’ parenting stress could be categorized into four potential profile types based on the distributional characteristics of the dimensions of parenting stress, naming them as high-stress type, middle-stress type, middle-stress-low interaction disorder type, and low-stress type, which is similar to the profile types derived from previous studies among groups of mothers [16], and supports Hypothesis 1. Mothers with high stress levels had the highest scores on all dimensions of parenting stress. Middle-stress mothers had moderate scores on all dimensions of parenting stress. Mothers with middle stress-low interaction disorder had moderate scores on the parental distress and difficult child dimensions, and scores were only low on the parent-child dysfunctional interaction dimension. Mothers with low stress levels had the lowest scores on all dimensions of parenting stress.

In addition to the dimensional characteristics of the four types, their distribution ratios also merit attention. Middle-stress mothers and high-stress mothers together accounted for the highest percentage (54.96%), suggesting that most adolescent mothers experienced relatively high parenting stress. This pattern may reflect the influence of traditional Chinese educational values and the growing pressure of academic competition in contemporary society [8], which may increase mothers’ parenting stress when their children enter adolescence. By contrast, about one quarter of mothers were classified into the low-stress profile (26.71%), who experienced low levels of parenting stress, likely because their higher educational attainment provides richer parenting knowledge and problem-solving skills [72]. The remaining mothers (18.32%) were in the middle-stress–low interaction disorder profile, who showed medium overall stress but low stress in parent–child interaction, possibly reflecting more effective interaction strategies and a relatively positive parent–child relationship [73]. Taken together, these four profiles reveal heterogeneous patterns of parenting stress among mothers of adolescents and provide additional evidence for how maternal parenting stress may affect adolescent development.

### 4.2. Mothers’ parenting stress profiles and adolescent problem behavior

The study results showed that, compared with the other three profiles, adolescents with low-stress mothers had significantly lower levels of problem behavior, whereas those with high-stress mothers had the highest levels. Moreover, all three higher-stress profiles were significantly and positively associated with adolescents’ problem behavior relative to the low-stress profile. These results are broadly consistent with previous findings [27] and provide partial support for Hypothesis 2. It has been found that high-intensity parenting stress often migrates to mothers’ parenting behaviors and emotional expressions, causing them to develop negative thoughts or behaviors [21], which in turn leads to detached or avoidant responses to their children [22]. This creates an unhealthy and high-pressure family environment [23]. At the same time, adolescents in this high-pressure environment are more likely to internalize the perceived stress into their own emotions and eventually develop high levels of problematic behaviors [24].

However, no significant difference was found in problem behaviors between adolescents whose mothers were in the middle-stress profile and those in the middle-stress–low interaction disorder profile, suggesting that variation in parent–child dysfunctional interaction may not exert a strong direct effect on adolescents’ problem behaviors. This result diverges from previous research [27] and does not fully support Hypothesis 2. One possible explanation is that, during adolescence, the primary focus of social interaction shifts from the family to the school context [74], so the quality of mother–adolescent interactions may play a relatively smaller role in problem behaviors. Overall, these findings imply that only more severe or persistent dysfunction in mother–adolescent interactions is likely to substantially contribute to adolescents’ problem behaviors.

Parents often need to focus on the impact of their own parenting behaviors on adolescents [28]. According to the theory of emotional contagion, mothers with high parenting stress tend to experience stronger negative affect and more internalizing symptoms, including depressive and anxious symptoms. In the process of getting along with the child, the mother’s negative emotions will cause the child to be in a state of long-term emotional sensitivity and a lack of security in the outside world, thereby increasing internalizing symptoms such as anxiety and fear [75]. Regarding adolescents’ externalizing problem behaviors, empirical studies have consistently shown that maternal parenting stress significantly predicts adolescents’ externalizing problems [76]. Under China’s exceptional traditional concept of “hope one’s children will have a bright future” and the pressure of social competition, the pressure on parents to raise children is high [8]. Thus, exploring how different potential profiles of maternal parenting stress act on adolescents’ levels of problematic behaviors is of particular relevance to adolescent development.

### 4.3. Mediating effects of mothers’ work-family conflict

The results of the relative mediation analyses showed that, compared with the low-stress profile, the high-stress, middle-stress, and middle-stress–low interaction disorder profiles were associated with stronger work–to–family interference, which in turn predicted higher levels of adolescents’ problem behaviors, partially supporting Hypothesis 3. The above results suggest that work-family conflict diminishes mothers’ ability to cope with parenting stress by reducing their parenting involvement [39] while increasing mothers’ stress and emotional depletion during parent-child interactions [29,40], being harsher, irritable, and less warmly responsive to their children [77], leading to higher levels of problem behaviors in adolescents. The Work-Home Resources Model posits that an individual’s harmful emotional response resources, such as anxiety [78], negative emotions [79,80], and emotional depletion [81,82], adjust an individual’s total resources, which in turn affects the individual’s work-family relationship [45]. Thus, when mothers experience high levels of parenting stress, their resources are depleted and work–family conflict is more likely to occur.

Meanwhile, maternal work–to–family interference significantly and positively predicted adolescents’ problem behaviors, whereas family–to–work interference did not, a pattern that is partially consistent with previous findings [83]. A plausible explanation for the non-significant mediating role of family-to-work interference lies in domain-specificity. Prior meta-analytic evidence suggests that work-to-family interference is more strongly related to outcomes in the family domain. In contrast, family-to-work interference predominantly predicts work-domain outcomes such as job satisfaction or performance [84]. Because adolescent problem behavior is a family-domain outcome, stronger mediation via WIF than FIW is theoretically expected. In addition, within the Chinese cultural context, mothers often prioritize childcare responsibilities and can rely on extended family support, particularly grandparental care, to buffer the impact of family demands spilling over into the work domain [85]. Consequently, when family interferes with work, the adverse influence on children’s daily routines may be diluted. Taken together, these theoretical and cultural considerations provide a coherent explanation for why family-to-work interference did not significantly mediate the link between maternal parenting stress profiles and adolescent problem behavior.

Although relatively few empirical studies have directly examined the link between work–family conflict and children’s problem behaviors, work–family conflict has been widely recognized as an important determinant of the family environment and parenting behavior [86]. Most existing research has emphasized the beneficial aspects of mothers’ employment for families, such as increased access to resources and income [45]. However, according to the Scarcity Hypothesis, an individual’s time and resources are limited. In the case of work-family conflicts, mothers may have to give up some of their family activities, parent-child interactions, or work-related opportunities, resulting in fatigue, distress, or emotional withdrawal [87]. These maladaptive responses may be one way in which work-family disruptions for mothers affect the development of their adolescents. In conclusion, this study helps alleviate the disruptions to families caused by mothers’ work. These findings contribute to clarifying how maternal stress management and work–family balance strategies may help alleviate disruptions experienced by families and provide important references for designing additional social and policy support.

## 5. Limitations and future study directions

The present study took into account the differences that exist among individuals and used an individual-centered approach to categorize parenting stress among adolescent mothers, deepening the understanding of parenting stress among this group of mothers, as well as examining the mediating role of the dimensions of work-family conflict between various types of parenting stress among mothers and adolescent problem behaviors. However, the following shortcomings remain: First, participants were recruited through school-based convenience sampling from a single region, which reduces the representativeness of the sample and is not conducive to the external validity and generalizing the findings to other groups of mothers and adolescents in other regions or sociocultural contexts. Future studies should adopt more diverse and, where possible, probabilistic sampling strategies (e.g., multi-site or multi-stage sampling) to validate and extend the findings of this study further. Second, this study employed a cross-sectional design, which was not conducive to exploring whether the type of parenting stress among mothers changes over time and its potential impact on the level of adolescent problem behaviors. Third, although the SDQ total difficulties scale and the other instruments used in this study showed acceptable internal consistency and their confirmatory factor analyses supported the expected factor structures, several difficulty subscales and some fit indices were only in the moderate rather than excellent range. These psychometric constraints may have introduced measurement error and attenuated the associations between maternal stress profiles, work–family conflict, and adolescents’ problem behaviors. Future research could replicate these findings using instruments or revised scales with stronger psychometric properties.

## 6. Conclusion

(1) There are four latent types of parenting stress in mothers: high-stress type, middle-stress type, middle-stress-low interaction disorder type, and low-stress type. (2) The level of problem behavior of children of low-stress mothers was significantly lower than that of children of mothers in the other three profiles. The level of problem behaviors of children of high-stress mothers was significantly higher than that of children of mothers in the other three profiles. However, the difference between the level of problem behaviors of children of mothers in the middle-stress type and the middle-stress-low interaction disorder type was not significant. (3) Compared to the low-stress type, the high-stress type, middle-stress type, and middle-stress-low interaction disorder type all significantly and positively predicted adolescents’ problem behaviors. (4) Using the low-stress type as the reference group, interference with the family from the mother’s work significantly mediated the relationship between the other three maternal parenting stress types and adolescent problem behaviors.

These findings have important practical implications. First, they suggest that intervention programs should focus on reducing maternal parenting stress and providing psychological support to mothers, thereby preventing the emergence of adolescent problem behaviors. Second, considering the significant mediating role of work–family conflict, it is necessary to promote family-friendly workplace policies (e.g., flexible working arrangements, parental leave) and strengthen social support systems to help mothers balance their dual roles. Finally, the results highlight the need for parent education programs that emphasize effective parent–child interaction strategies, which may alleviate the adverse effects of high parenting stress on adolescents’ development. Beyond these practical implications, this study contributes to the literature by identifying distinct maternal parenting stress profiles in the Chinese cultural context and clarifying their links with adolescent problem behaviors through work–family conflict. Future research could further validate these findings using longitudinal designs and intervention-based approaches.

## Supporting information

S1 File Supplementary demographic comparisons, including independent-samples t tests (adolescent gender; number of children in the family) and one-way ANOVAs (maternal age; maternal educational level; family structure; working hours; monthly income), with LSD post-hoc comparisons where applicable. Notes: *p < 0.05, **p < 0.01, ***p < 0.001. Abbreviation: LSD, least significant difference.
(DOCX)

S2 File Checklist. (DOC)
