# Supplementary material for: Relationship between parenting stress profiles, work-family conflict, and adolescent problem behavior: Variable-centered and person-centered approaches
Source: PLoS One. 2026 Feb 25;21(2):e0340958. doi: 10.1371/journal.pone.0340958 (PMC12935267; doi:10.1371/journal.pone.0340958)
Supplement: S1 File — Notes: *p < 0.05, **p < 0.01, ***p < 0.001. Abbreviation: LSD, least significant difference. (DOCX) [file pone.0340958.s001.docx]

| **Table S1**  Results of independent samples t-test. | | | | | |
| --- | --- | --- | --- | --- | --- |
| Classification | | *N* | *M* | *SD* | *t* |
| Adolescent Gender | Boy | 416 | 9.58 | 4.494 | 3.623*** |
|  | Girl | 430 | 8.49 | 4.207 |  |
| Number of children in a family | One child | 274 | 9.83 | 4.242 | 3.715*** |
|  | Two children and above | 572 | 8.64 | 4.399 |  |
| **Note:** ****p* < 0.001. | | | | | |

| **Table S2**  Results of one-way analysis of variance. | | | | | | |  |
| --- | --- | --- | --- | --- | --- | --- | --- |
| Classification | | *N* | *M* | *SD* | *F* | LSD | |
| Maternal Age | 35-40 years old | 95 | 11.24 | 4.166 | 14.552*** | ①>②③ | |
|  | 41-45 years old | 592 | 8.82 | 4.364 |  |  |  |
|  | 46-50 years old | 159 | 8.47 | 4.208 |  |  |  |
| Educational Level of Mothers | Secondary school and below | 314 | 9.61 | 4.697 | 5.713** | ①②>③④ | |
|  | College or correspondence undergraduate | 199 | 9.34 | 4.053 |  |  |  |
|  | Full-time undergraduate degree | 297 | 8.36 | 4.196 |  |  |  |
|  | Master's degree or above | 36 | 7.67 | 3.891 |  |  |  |
| Family Structure | Divorced or single-parent families | 86 | 9.97 | 4.557 | 1.657 | - | |
|  | Nuclear family | 515 | 8.88 | 4.444 |  |  |  |
|  | The big family of three generations | 219 | 8.94 | 4.140 |  |  |  |
|  | Other family forms | 26 | 9.54 | 4.366 |  |  |  |
| Working Hours | Full-time mothers | 162 | 9.65 | 4.330 | 3.656* | ①③>② | |
|  | 8 hours or less | 252 | 8.49 | 4.188 |  |  |  |
|  | 8 hours or more | 432 | 9.10 | 4.829 |  |  |  |
| Month Income | RMB 6,000 and below | 186 | 9.51 | 4.509 | 1.098 | - | |
|  | RMB 6,000 to 10,000 | 265 | 9.03 | 4.421 |  |  |  |
|  | RMB 10,000 to 15,000 | 194 | 8.92 | 4.522 |  |  |  |
|  | RMB 15,000 to 20,000 | 100 | 8.96 | 4.119 |  |  |  |
|  | RMB 20,000 and above | 101 | 8.41 | 3.990 |  |  |  |
| **Notes:** **p* < 0.05, ***p* < 0.01, ****p* < 0.001.  **Abbreviation:** LSD, least significant difference. | | | | | | |  |
